# Sertraline Pre-Treatment Attenuates Hemorrhagic Transformation Induced in Rats after Cerebral Ischemia Reperfusion via Down Regulation of Neuronal CD163: Involvement of M1/M2 Polarization Interchange and Inhibiting Autophagy

**DOI:** 10.1007/s11481-023-10093-8

**Published:** 2023-11-13

**Authors:** Shimaa K. Mohamed, Amany A. E. Ahmed, Abeer Elkhoely

**Affiliations:** Department of Pharmacology and Toxicology, Faculty of Pharmacy, Helwan University, Ein Helwan, Cairo, 11795 Egypt

**Keywords:** Sertraline, Cerebral I/R, Hemorrhagic transformation, M2 phenotype, MMP-9, HO-2, Autophagy, Apoptosis

## Abstract

Cerebral ischemia reperfusion (I/R) is one of the neurovascular diseases which leads to severe brain deterioration. Haemorrhagic transformation (HT) is the main complication of ischemic stroke. It exacerbates by reperfusion, causing a more deleterious effect on the brain and death. The current study explored the protective effect of sertraline (Sert) against cerebral I/R in rats by inhibiting HT, together with the molecular pathways involved in this effect. Forty-eight wister male rats were divided into 4 groups: Sham, Sert + Sham, I/R, and Sert + I/R. The ischemic model was induced by bilateral occlusion of the common carotid artery for 20 min, then reperfusion for 24 h. Sertraline (20 mg/kg, p.o.) was administrated for 14 days before exposure to ischemia. Pre-treatment with Sert led to a significant attenuation of oxidative stress and inflammation. In addition, Sert attenuated phosphorylation of extracellular regulated kinases and nuclear factor kappa—p65 expression, consequently modulating microglial polarisation to M2 phenotype. Moreover, Sert prevented the hemorrhagic transformation of ischemic stroke as indicated by the notable decrease in neuronal expression of CD163, activity of Heme oxygenase-2 and matrix metalloproteinase-2 and 9 levels. In the same context, Sert decreased levels of autophagy and apoptotic markers. Furthermore, histological examination, Toluidine blue, and Prussian blue stain aligned with the results. In conclusion, Sert protected against cerebral I/R damage by attenuating oxidative stress, inflammation, autophagy, and apoptotic process. It is worth mentioning that our study was the first to show that Sert inhibited hemorrhagic transformation.

**Graphical Abstract:**

The protective effect of sertraline against injury induced by cerebral ischemia reperfusion via inhibiting Hemorrhagic transformation.

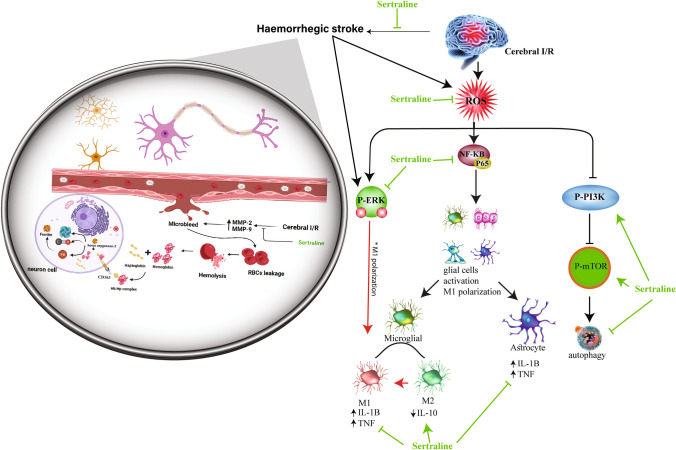

**Supplementary Information:**

The online version contains supplementary material available at 10.1007/s11481-023-10093-8.

## Introduction

Ischemia occurs due to obstruction of the blood vessels and leads to a decrease in oxygen and glucose required for cellular demands, thus causing tissue injury. On the other hand, reperfusion restores the blood flow, an event which leads to a more aggressive deterioration to the tissue. Cerebral I/R injury takes place after partial restoration of blood flow (Wanchao et al. 2018; Wang et al. 2020). Moreover, it is significant to mention that ischemic stroke is one of the detrimental causes of disability worldwide (Wu et al. 2017).

Restoration of blood flow to brain tissue after ischemia results in a tremendous reactive oxygen species (ROS) production, which causes upregulated expression of inflammatory mediators (Jurcau and simion 2022). This inflammatory process happens at 3 levels as follows: acute stage (the first hours of incidence of stroke), in which microglia damage the degenerated cells and leukocytes infiltrate, commonly neutrophils; the subacute stage, which relies upon the release of the inflammatory mediators; late-stage depends on astrocyte and microglial cell reparatory role (Jurcau and simion 2022).Consequently, due to the inflammatory status of the brain tissue, the apoptotic cascade is activated either directly or indirectly. Finally, these destructive manifestations disrupt the blood membrane barrier, edema, and hemorrhagic transformation (HT) (Jurcau and simion 2022).

Microglial cells are native immune cells in the central nervous system which check the brain parenchyma. According to the type of stimulus, microglial cells are activated and switched between two polarisation phenotypes, the phenotype (M1) and the alternative phenotype (M2) (Zhang et al. 2019; Yu et al. 2020). Microglial cells can take an inflammatory or pro-inflammatory role via switching between M1 and M2-activated phenotypes, affecting the progression of ischemic stroke. The microglial cell shifts to the M1 phase during cerebral I/R, due to the release of inflammatory mediators, ROS, DAMPs, and neuromodulators from degenerated neurons in the ischemic core and activation of nuclear factor kappa-β (Fernandez-Lizarbe et al. 2009). Cytotoxic factors such as inflammatory mediators and nitric oxide are released from the M1 phenotype, which also disrupts the blood–brain barrier (BBB), increases its permeability, and engulfs vascular endothelial cells, causing a breakdown of this barrier after stroke (Xu et al. 2020).On the other hand, M2 polarisation enhances anti-inflammatory factors, eliminates debris, induces angiogenesis, and repairs peripheral injured neurons (Dong et al. 2021). Also, the M1 phenotype has a role in amplifying necroptosis of endothelial cells and disruption of BBB following ischemic stroke (Jiang et al. 2020). All these together cause HT that is detected by numerous markers such as CD 163, which acts as a scavenger receptor on neurons for haemoglobin (Hb), allowing safe getting rid of Hb without toxic effects that worsen brain status after stroke (Leclerc et al. 2018).

Autophagy is a fundamental biological process that causes the breakdown and reprocessing of cellular constituents and consequently underlies many physiological and pathological conditions such as diabetes, neurodegenerative diseases, and obesity. It is reported that autophagy participates in ischemic diseases such as myocardial infarction, focal ischemia, global ischemia, cerebral I/R injury, and hypoxic ischemia (Sun et al. 2020; Zhang et al. 2020).

Selective serotonin reuptake inhibitors (SSRI) are resorted to for managing many psychological diseases, mainly anxiety disorders. Sertraline (Sert) is one of the most prescribed SSRI drugs for managing several conditions, such as anxiety, depression, and obsessive–compulsive disease (Atli et al. 2017). Previous studies documented the anti-inflammatory effect of Sert through decreasing production of cyclooxygenase-2, TNF-α and IL-1β (Baharav et al. 2012; Sitges et al. 2014). Also, Sert exhibited antioxidant, antiapoptotic and neurogenic effects (Ozturk et al. 2013).

It is noteworthy that only a few studies have investigated hemorrhagic transformation as a secondary impact of cerebral stroke on brain injury and how it increases the rate of morbidity and mortality. Hence, our study was established aiming to assess the protective potential of Sert against cerebral I/R-induced brain injury in rats. In parallel, the study focused on investigating the molecular pathways impacted by Sert, specifically microglial polarisation and autophagy, along with the relevance of these molecular pathways to the inhibition of the transformation of ischemic stroke to hemorrhagic stroke.

## Materials and Methods

### Drugs

Sert was kindly granted by the Egyptian Company for Chemicals and Pharmaceuticals, Cairo, Egypt. All solvents and chemicals were of analytical grade.

### Animals

Forty-eight Wister male rats weighing 180 to 200 gm were purchased from the breeding unit of the Egyptian Organization of Biological Products and Vaccines. Approval of the experimental protocol was taken from the ethical committee at the Faculty of Pharmacy, Helwan University (Protocol Number: 05A2021). Randomly, the rats were distributed (4 rats per cage) at a controlled temperature (22 ± 2 ^0^c) and environmental settings. A week before the experiment, they were habituated with free access to a standard pellet diet and drinking water ad libitum.

### Experimental Groups and Treatment

Randomly, animals were distributed into 4 groups (12rats/group). The first group (Sham group) and the second group (Sert + Sham group) were exposed to the identical surgical steps of cerebral I/R injury without occlusion of the carotid artery and received saline (1 ml/kg; p.o.) and Sert (20 mg/kg; p.o.) respectively, for 14 days. The third group (I/R group) and the fourth group (Sert + I/R group) received saline (1 ml/kg; p.o.) and Sert (20 mg/kg; p.o.) respectively, for 14 days (Shin et al. 2009) followed by cerebral I/R injury. Following cerebral I/R injury, the rats were decapitated, and the brain was excised to be utilised for further studies.

#### Induction of Cerebral I/R

Twenty-four rats were randomly chosen, fasted overnight, and anesthetised using chloral hydrate (360 mg/kg, i.p) at the time of surgery. For induction of ischemia, the carotid arteries were bilaterally ligated for 20 min. Afterwards, the circulation was restored through the de-ligation of arteries, and reperfusion was allowed for 24 h (Jin et al. 2019).

After 24 h reperfusion, rats were decapitated; brains were immediately isolated from the skull on ice, and rinsed with ice-cold phosphate-buffered saline (PBS). The samples were distributed as follows:

The left hemisphere (6 per group) was homogenised in PBS to get 10% W/V homogenate, then they were stored at -20 ^0^C. Homogenates were exposed to two freeze–thaw cycles to disturb the cell membrane. After that, the homogenate was centrifuged at 4^0^ C for 5 min at 5000 rpm, then the supernatants were set for biochemical analysis, while the right hemisphere (6 per group) was set for western blot measures.

The other six brains from each group were fixed for histopathological investigation.

### Biochemical Assay

Glutathione (GSH), nitrate/nitrite and malondialdehyde (MDA) levels were estimated in whole brain tissue using kits; Bio-Vision (Milpitas, CA, USA), Cayman chemical (Ann Arbor, MI, USA) and Sigma-Aldrich Co. (St. Louis, MO, USA) respectively. Furthermore, ELISA kits were utilised for the estimation of BcL_2_, Bax, TNF‐α, matrix metalloproteinase 2,9 (MMP-2,9) and IL‐10 levels (CUSABIO, Wuhan, China), IL-1β (MyBioSource, San Diego, CA, USA), Heme oxygenase-2 (HO-2) (Abbexa, Cambridge, United Kingdom) and caspase-3(Bio-Vision, Milpitas, CA, USA). All steps were accomplished as mentioned by the manufacturer. Data were calculated relative to the brain tissue protein content.

### Western Blot Analysis

RIPA lysis buffer and Bradford Protein Assay Kit were used for protein determination from brain tissues (Bio Basic Inc., Markham, Ontario, Canada). The separated proteins were loaded and incubated on the PVDF membrane with primary antibodies for Beclin 1 (# PA5-20172), p-ERK (#44-680G), p-mTOR (#44-1125G), p-PI_3_K (# PA5-99367), ERK ( #13–6200), mTOR (# AHO1232), LC_3_ A (#PA5-23180), LC_3_ B (#PA1-16930), NF‐κB (p65) (# 51–0500), and β-actin (# MA5-15739) (Thermo Fisher Scientific Inc., Waltham. MA, USA) at 4°C. Following washing, blots were incubated with peroxidase‐labeled anti‐rabbit secondary antibodies at 37°C for 1 h. protein Bands intensity was detected using the chemiluminescent substrate (ClarityTM Western ECL substrate—Bio-Rad, TNC, USA), and a CCD camera-based imager. Analysing the images was done using Chemi Doc MP Imager and then normalised by β-actin.

### Histopathological Examination and Immunohistochemistry Analysis

#### Histological Examination

Brain tissues were fixed in 10% neutral buffered formalin for 72 h. Afterwards, all specimens were washed with tap water, dehydrated in serial dilutions of ethanol, cleared in Xylene, and put into Paraplast tissue *(Leica biosystems).* Brain sections (sagittal) of 5 microns thickness were fitted on glass slides to examine cortical and hippocampal regions in various specimens. Brain tissues were then stained with Hematoxylin and Eosin (H & E) to be investigated with a light microscope. Also, other brain tissues were stained with Toluidine blue and Prussian blue to detect the mean count of intact, injured neurons and the number of ferric ion deposits, respectively. Fixation and staining of the samples were accomplished consistently with the standard procedures and protocols (Culling 2013).

#### Immunohistochemistry Staining and Analysis

Brain specimens were implanted in paraffin, and sections were cut to a thickness of 5 microns. Immunohistochemical staining was carried out following instructions mentioned by the manufacturer. Deparaffinisation and retrieving tissue sections were done, followed by soaking in 0.3% H_2_O_2_ for 20 min. Afterwards, brain tissues were treated with Anti– Glial Fibrillary Acidic Protein antibody as a marker for activated astrocytes (Cat. No. 13–0300- thermo scientific Co. 1:100), anti CD86 primary antibody as a marker for M1 microglial phenotype (bs-1035R, BIOSS USA – 1:150), anti CD163 antibody as a marker for M2 microglial phenotype (GeneTex. GTX35247 1:100) and Anti-Iba1 antibody as a marker for activated microglial cells (ab108539-Abcam-1:100) overnight at 4 °C. Following washing, brain tissues were incubated with a secondary antibody HRP Envision kit (DAKO) for 20 min; after that, they were incubated with diaminobenzidine (DAB) for a time interval of 15 min. Following washing, hematoxylin was used to counterstain tissues. Later, tissues were dehydrated and cleared in xylene for microscopic scanning of the cortical and hippocampal (CA1) region.

#### Histological Analysis

According to (Abbas et al. 2021), 6 non-overlapping fields in CA1 and cerebral cortex were chosen randomly in each sample and examined for detecting relative area percentage of immunohistochemical expression of GFAP; mean reactive microglial cells counts for CD163, CD86 and Iba-1 in immunohistochemically stained sections. Besides, the mean count of intact neurons in CA1 and cortex in toluidine blue stained tissue sections was performed. Also, the number of Ferric ion deposits was detected by Prussian blue stain in CA1 and cerebral cortex.Lastly, the optical density (OD) of CD163 expressed in neuron cells of CA1 and cortex was measured.

The Leica Application module linked to the imaging system (Leica Microsystems GmbH, Germany) was used to examine and obtain data.

### Statical Analysis

Results are expressed as (mean ± standard error) by using GraphPad 9 software. Significance between groups was detected using one-way analysis of variance (ANOVA) and then Tukey’s multiple comparison test. Groups were regarded as significantly different from each other, if p value was at least < 0.05.

## Results

### Sert Decreased Oxidative Stress of Cerebral I/R in Rats

**Fig. 1:**
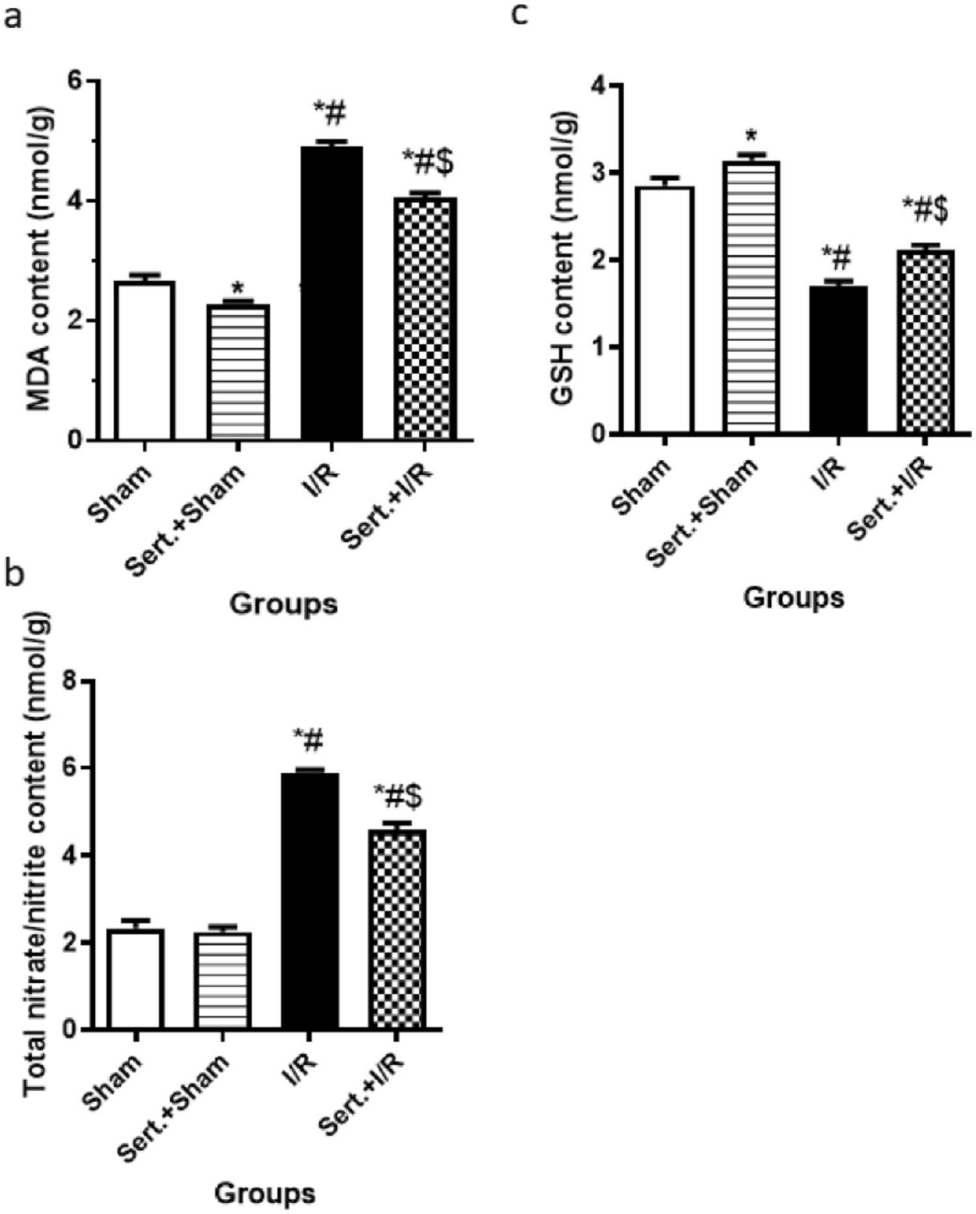
**a-c** Effect of Sert on oxidative stress of cerebral I/R in rats: **a** MDA, **b** Total nitrate/nitrite, **c** GSH content. Results were illustrated as mean ± SE (n = 6). *: significant from Sham group (p < 0.05), # significant from Sert + Sham group (p < 0.05), $: significant from I/R group (p < 0.05). Test of significance was accomplished using one way ANOVA, afterwards, Tukey’s multiple comparison test was achieved. MDA: Malonaldehyde, GSH: Glutathione

As illustrated in Fig. 1(a–c), cerebral I/R rats showed a significant increase of oxidative stress markers as detected by the remarkable increase of MDA, total nitrate/nitrite contents by 77%, 1.5-fold and notable decrease of GSH level by 40.38% in comparison with sham group respectively at p < 0.05. On the contrary, pre-treatment with Sert resulted in a significant decrease of MDA, and total nitrate/nitrite contents by 13, and 22.6%, respectively at p < 0.05 and an elevation of GSH level by 28% as relevant to the ischemic group at p < 0.05.

### Effect of Sert on Astrocytes and Microglial Cells of Cerebral in I/R Rats

**Fig. 2:**
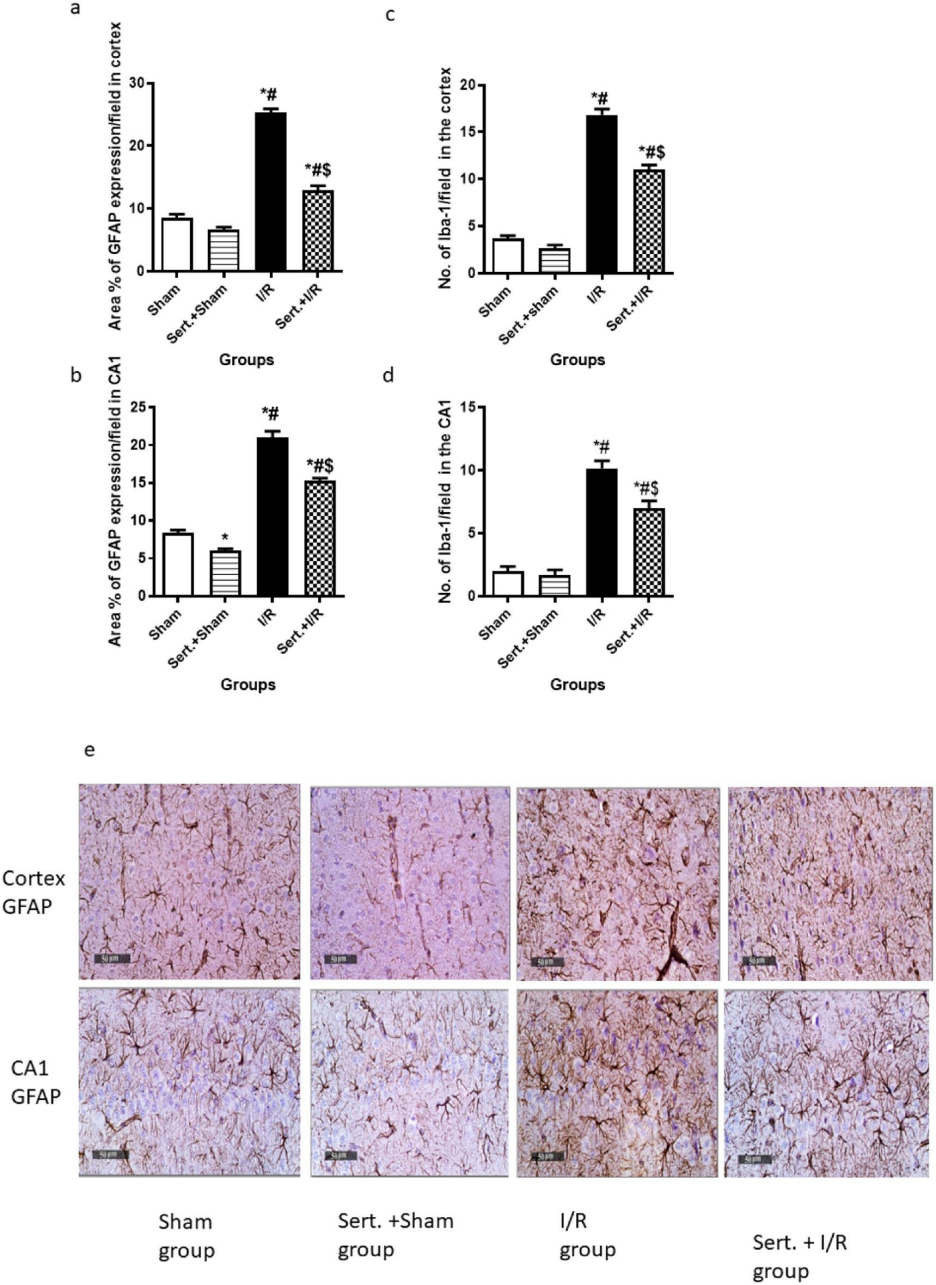

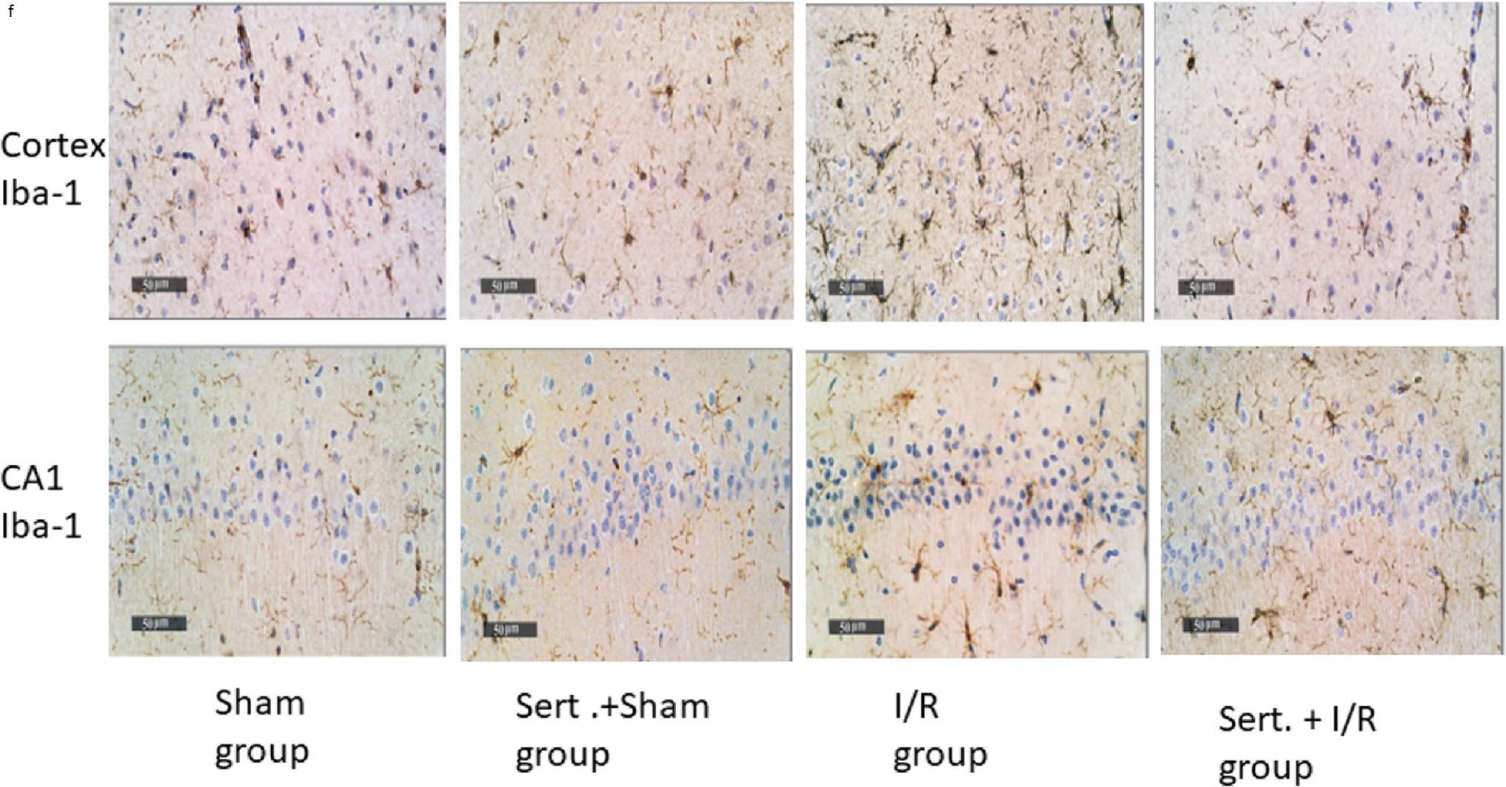
**a-f** Effect of Sert on activation of the microglial cells and astrocytes of cerebral I/R in rats: **a**-**d** GFAP, Iba-1 quantitative expression in cortex and CA1, **e**–**f** Representative photomicrography showing an increase of GFAP, and Iba-1 expression in both cortex and CA1 in I/R group as compared to sham group. The illustrated photos were magnified by × 400, with scale bars = 50 μm. Results were illustrated as mean ± SE (n = 6). *: significant from Sham group (p < 0.05), # significant from Sert + Sham group (p < 0.05), $: significant from I/R group (p < 0.05). Test of significance was accomplished using one way ANOVA, afterwards Tukey’s multiple comparison test was achieved. GFAP: Glial fibrillary acidic protein, Iba-1: Ionised calcium binding adaptor molecule 1

As illustrated in Fig. 2(a–f), cerebral I/R activated the microglial cells and astrocytes as confirmed by the notable increment of GFAP and Iba-1 expression in both cortex and hippocampus by 2, 1.5,3.6, 4-fold, respectively, as compared to a sham group at p < 0.05. Pre-treatment with Sert caused a remarkable decrease of GFAP expression in cortical and CA1 regions by 49.1%, 27%, respectively at p < 0.05 and a notable decline of Iba-1 expression in cortex and CA1 by 34.7%, 31.2% at p < 0.01, respectively in relevance with the ischemic group.

### Sertraline Decreased Inflammation of Cerebral I/R in Rats:

**Fig. 3:**
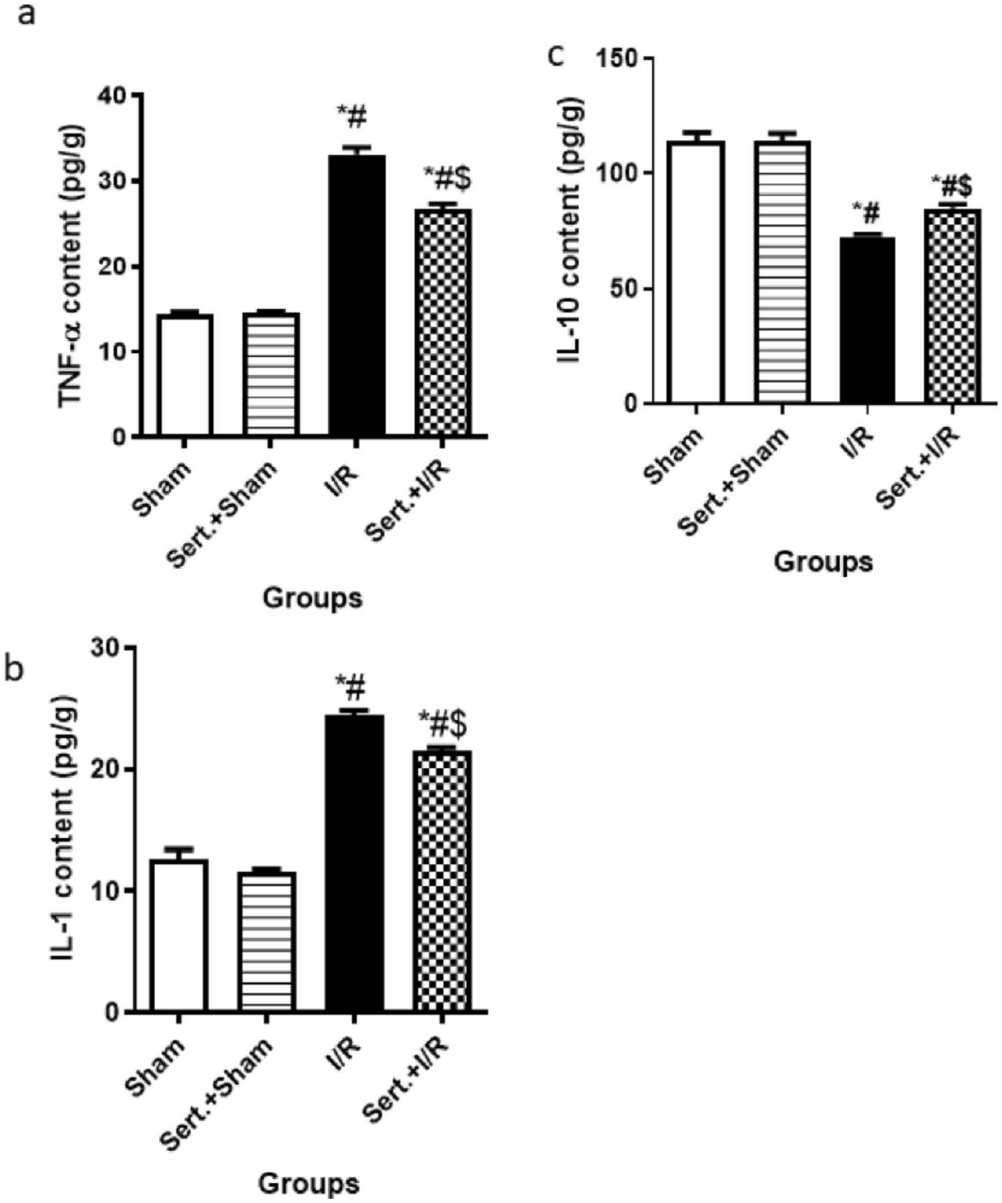
**a-c** Effect of Sert on inflammation predisposed by cerebral I/R in whole brain tissues of rats: **a** TNF-α, **b** IL-1, and **c** IL-10 content. Results were illustrated as mean ± SD (n = 6). *: significant from Sham group (p < 0.05), # significant from Sert + Sham group (p < 0.05), $: significant from I/R group (p < 0.05). Test of significance was accomplished using one way ANOVA, afterwards Tukey’s multiple comparison test was achieved. IL: Interleukin, TNF-α: Tumor necrosis factor-alpha

As elucidated in Fig. 3(a–c), cerebral I/R in rats led to remarkable elevation of inflammatory mediators, TNF-α, IL-1 contents by 1.3-fold, 93.4%, respectively, and a significant decrease of IL-10 content by 36.2% as relevant to a sham group at p < 0.05. Interestingly, pre-treatment with Sert reversed all the previously mentioned findings as shown by the significant decrease of TNF-α, and IL-1 levels by 19, and 12% respectively at p < 0.05 and enhancement of IL-10 content by 17% at p < 0.05 as relevant to ischemic group.

### Effect of Sert on Phenotype of Microglial Cell of Cerebral I/R in Rats via Involvement of NF-ĸβ, and p-ERK

**Fig. 4:**
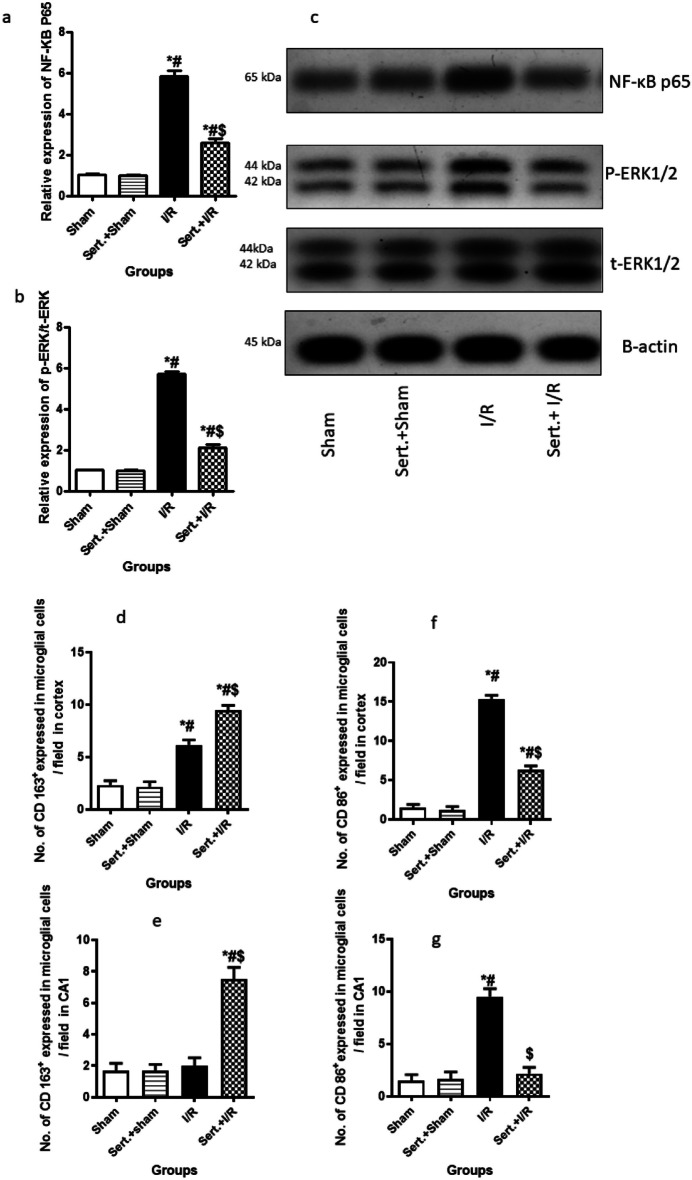

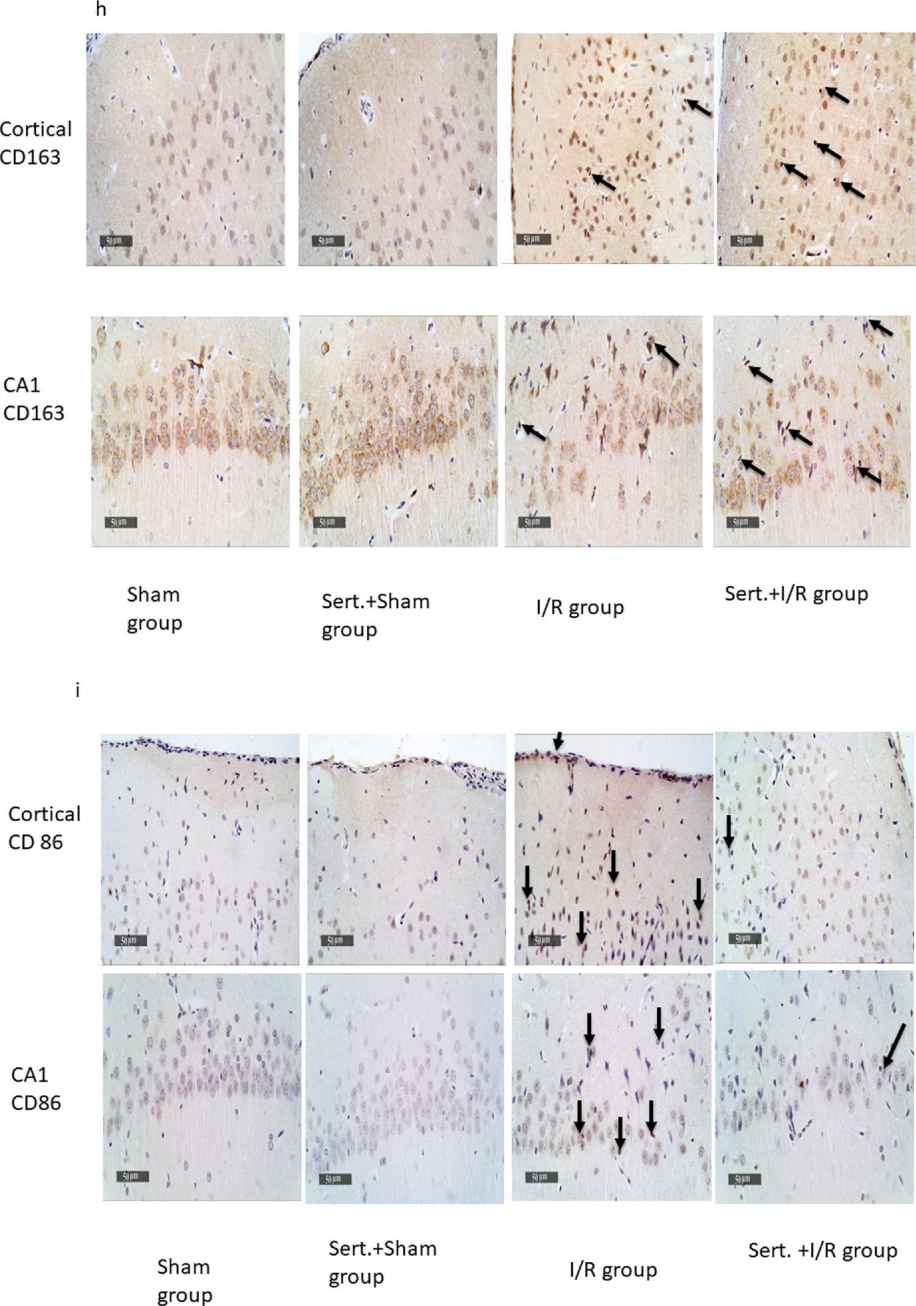
**a-i**: Effect of Sert on microglial/macrophage polarisation of cerebral I/R in rats: **a**-**b** Quantitative expression of NF-ĸBp65, p-ERK/t-ERK, **c** Representative western blot bands of NF-ĸb p65 and p-ERK expression in different treatment groups, **d**-**g** Quantitative CD 163 and CD 86 expression in both cortical and CA1 regions for different treatment groups, **h**-**i** Representative photomicrography displaying CD86 and CD 163 expression in both regions, cortex and CA1 for different treatment groups. The illustrated photos were magnified by × 400, with scale bars = 50 μm. Results were illustrated as mean ± SE (n = 6). *: significant from Sham group (p < 0.05), # significant from Sert + Sham group (p < 0.05), $: significant from I/R group (p < 0.05). Test of significance was accomplished using one-way ANOVA, afterwards Tukey’s multiple comparison test was achieved. CD: Cluster of differentiation, ERK1/2: Extracellular signal related kinases, NF-ĸBp65: Nuclear factor kappa B

As illustrated in Fig. 4(a–i), cerebral I/R in rats resulted in a notable increase of NF-ĸB P65 and p-ERK expression by 4.6, and 4.7-fold, respectively at p < 0.05. This led to M1 phenotype activation as indicated by increased expression of CD 86 in both cortex and CA1 region by 9.2, and 5.3-fold, respectively in comparison with the sham group, at p < 0.05. Conversely, pre-treatment with Sert significantly decreased expression of NF-ĸB P65 and p-ERK by 55.8,62.8%, respectively at p < 0.05 as compared to I/R group, hence shifting microglial cells to M2 phase as evidenced by a remarkable decrease of CD 86 by 58.7, 77.2%, respectively and increase of CD 163 expression in both cortical and CA1 regions by 54%, 2.8-fold, respectively in comparison with the ischemic group respectively at p < 0.05.

### Effect of Sert on Ischemic Stroke Transformation to Haemorrhagic Stroke of Cerebral I/R in Rats

**Fig. 5:**
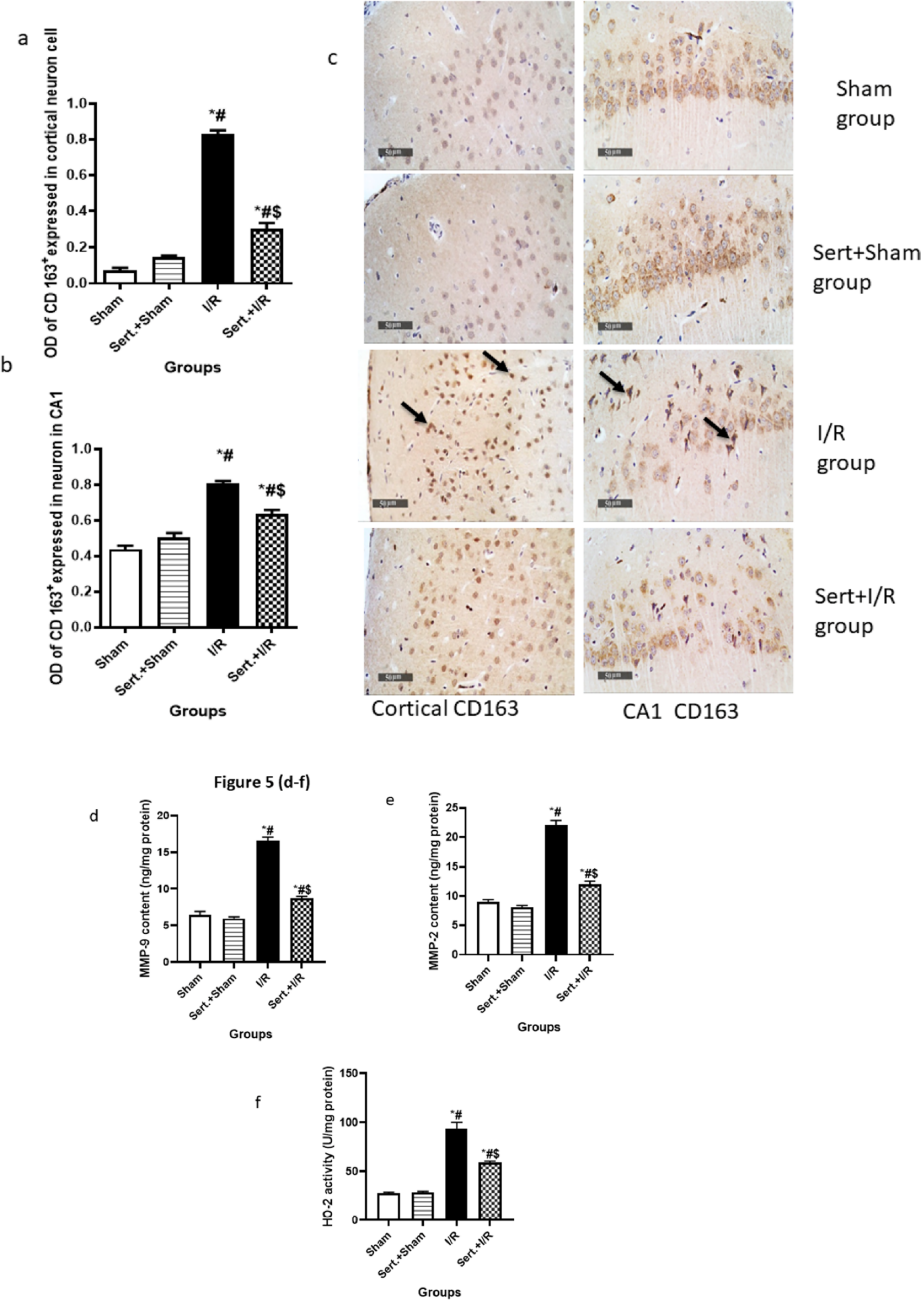

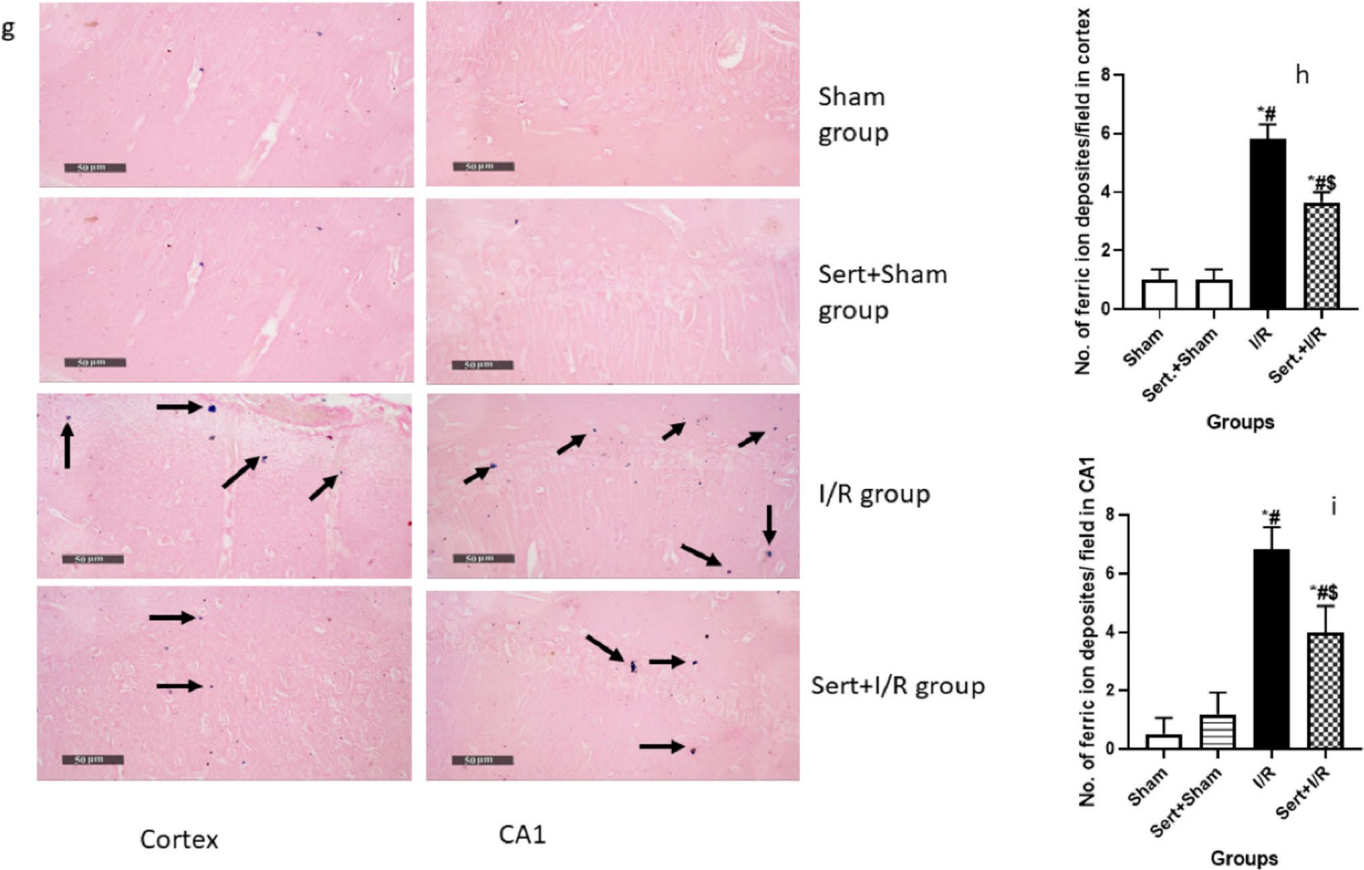
**a-i** Effect of Sert on the transformation of ischemic stroke to haemorrhagic of cerebral I/R in rats: **a**-**b** Quantitative neuronal CD 163 protein expression in both cortex and CA1, **c** Representative photomicrography displaying the expression of neuronal CD 163 in cortical and CA1 regions for different treatment groups, **d**-**e** level of MMP-9, MMP-2 for the different treated group, **f** activity of heme oxygenase-2 for all treated groups, **g** representative photomicrography for ferric ion deposits in both cortex and CA1 region, and **h**-**i** the number of Ferric ion deposits in both cortex and CA1region. The illustrated photos were magnified by × 400, with scale bars = 50 μm. Results were illustrated as mean ± SE (n = 6). *: significant from Sham group (p < 0.05), # significant from Sert + Sham group (p < 0.05), $: significant from I/R group (p < 0.05). Test of significance was accomplished using one way ANOVA, afterwards Tukey–Kramer multiple comparison test was achieved. CD: Cluster of differentiation

As elucidated in Fig. 5(a–i), cerebral I/R in rats induced transformation of ischemic stroke into haemorrhagic stroke as evidenced by a remarkable increment of CD 163 expression in neuron cells of both cortex and CA1 region by 10.6- fold, 84%, respectively in comparison with the sham group at p < 0.05. Contrarily, Sert remarkably halted haemorrhagic transformation (HT) by significantly decreasing the expression of CD 163 in neuron cells of both regions, cortex and CA1 by 64, 21% as compared to the ischemic group at p < 0.05, respectively. This was evidenced by measuring the levels of MMP-2,9, which are responsible for blood membrane barrier integrity. Cerebral I/R displayed an increment increase in the level of MMP-2,9 by 1.4, and 1.6-fold, respectively as compared to the sham group at p < 0.05. On the other hand, pre-treatment with Sert reversed that by a significant decrease in levels of MMP-2,9 by 45.3,47.3%, respectively as compared to the I/R group.

Moreover, it was found that the activity of HO-2 significantly increased in I/R by 2.4-fold compared to the sham group at p < 0.05. This is a clue for the presence of haemoglobin in the extracellular matrix between neurons due to haemorrhagic transformation. However, pre-treatment converted this situation by a significant decrease in the activity of HO-2 by 36.6% compared to the I/R group.

Finally, the previously mentioned results were further confirmed by the Prussian blue stain, which counts the number of ferric ion deposits in the field due to the lysis of red blood corpuscles (RBCs) and release of the heme group. In the I/R group, there was a significant increase in the number of ferric ion deposits in both cortex and CA1 by 4.8, and 12.6 -fold, respectively at p < 0.05 as compared to the sham group. On the other hand, pre-treatment with Sert decreased the number of ferric ion deposits in both cortex and CA1 by 37, and 36.6%, respectively at p < 0.05 as compared to the I/R group.

### Sert Decreased Autophagy of Cerebral I/R in Rats

**Fig. 6:**
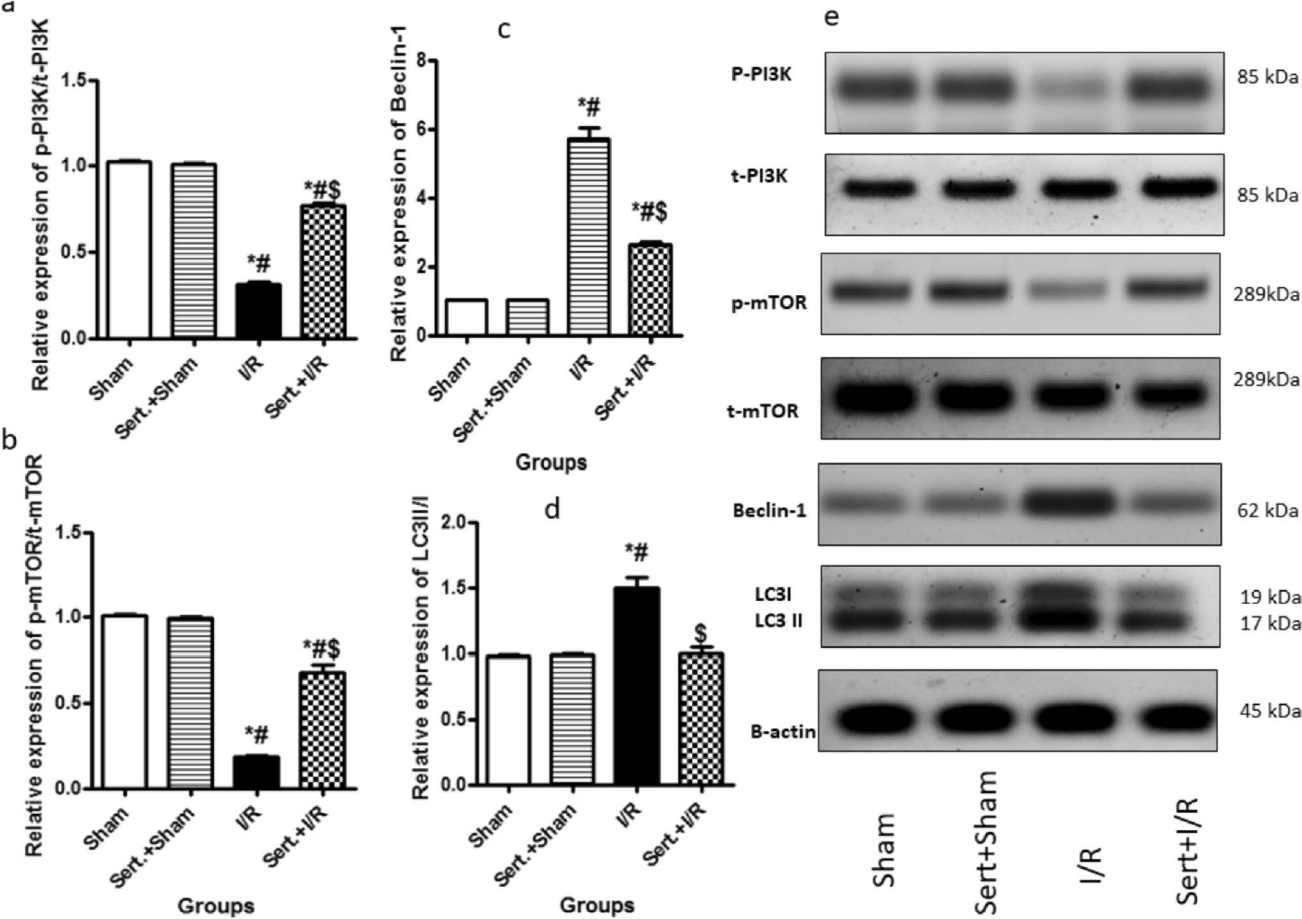
**a-e** Effect of Sert on autophagy of cerebral I/R in rats: a-d) Quantitative p-PI3K, p-mTOR, Beclin-1 and LC3II/I protein expression, e) Representative western blot bands showing expression of p-PI3K, p-mTOR, Beclin-1 and LC3II/I in different treatment groups. Results were illustrated as mean ± SE (n = 6). *: significant from Sham group (p < 0.05), # significant from Sert + Sham group (p < 0.05), $: significant from I/R group (p < 0.05). Test of significance was accomplished using one way ANOVA, afterwards Tukey–Kramer multiple comparison test was achieved. p-mTOR: phosphorylated Mammalian target of rapamycin, p-PI3K: phosphorylated Phosphoinositide 3-kinases

As illustrated in Fig. 6(a–e), autophagy was enhanced in rats exposed to cerebral I/R as detected by the significant decline in p-PI3K, p-mTOR expression by 69, 82%, respectively and increase in expression of Beclin-1, LC-3II/I by 4.7-fold, 54%, respectively as compared by sham group at p < 0.05. Contrarily, pre-treatment with Sert remarkably suppressed autophagy as manifested by the significant decrease of Beclin-1, LC3-II/I expression by 54.3, 34%, respectively and increased expression of p-PI3K, p-mTOR by 1.45, 2.7-fold, respectively as relevant to I/R group at p < 0.05.

### Effect of Sert on Bax, Bcl-2, Caspase-3 Contents and Neuronal Density of Cerebral in I/R Rats

**Fig. 7:**
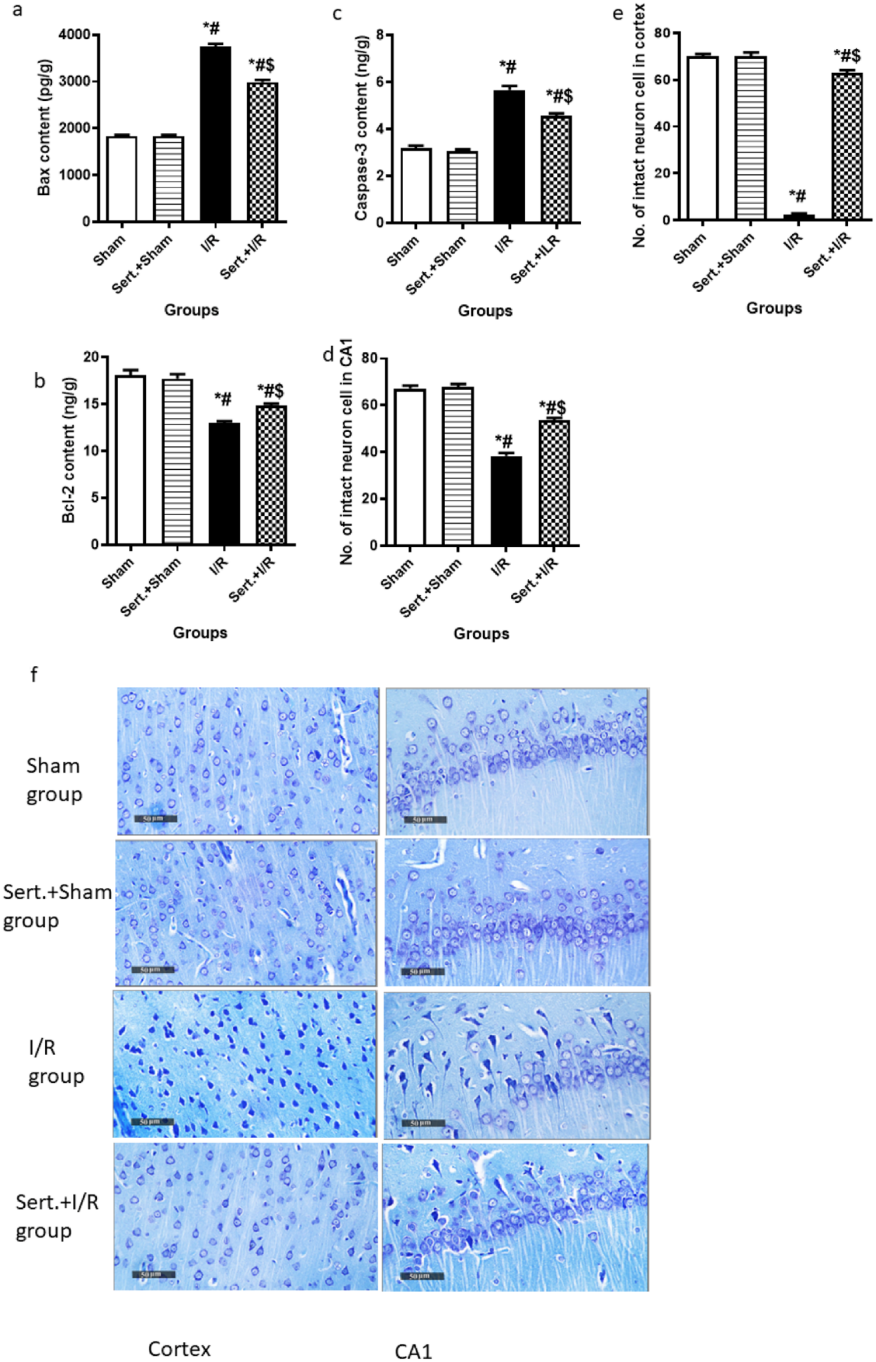
**a-f** Effect of Sert on Bax, Bcl-2, caspase-3 contents and neuronal density of cerebral I/R in rats: **a**-**e** Quantitative expression of Bax, Bcl-2, caspase -3, number of intact neurons in both region cortex and CA1, **f** Representative photomicrography displaying the number of intact neurons in both cortex, CA1 in different treatment groups after staining with toluidine blue. The illustrated photos were magnified by × 400, with scale bars = 50 μm. Results were illustrated as mean ± SE (n = 6). *: significant from Sham group (p < 0.05), # significant from Sert + Sham group (p < 0.05), $: significant from I/R group (p < 0.05).Test of significance was accomplished using one way ANOVA, afterwards Tukey’s multiple comparison test was achieved. Bcl-2: B-cell lymphoma 2, Bax: BCL2 Associated X

Figure 7(a–f) illustrates the apoptotic induction in cerebral I/R in rats as evidenced by the significant increment of Bax, and Caspase-3 contents by 1-fold, 78%, respectively, reduction of Bcl-2 level by 28.3% as well as decreased intact neuron cell number in both cortex and CA1 by 96.7,43% respectively as compared to the sham group at p < 0.05. However, pre-treatment with Sert induced neuronal survival as evidenced by a remarkable decrease of Bax, Caspase-3 levels by 20.4%,19.5%, increased content of Bcl-2 by 14.3%, and enhanced intact neuron cell number in both cortical and CA1 regions by 26-fold, 40%, respectively at p < 0.05.

### Effect of Sert on Histological Changes of Cerebral I/R in Rats

**Fig. 8:**
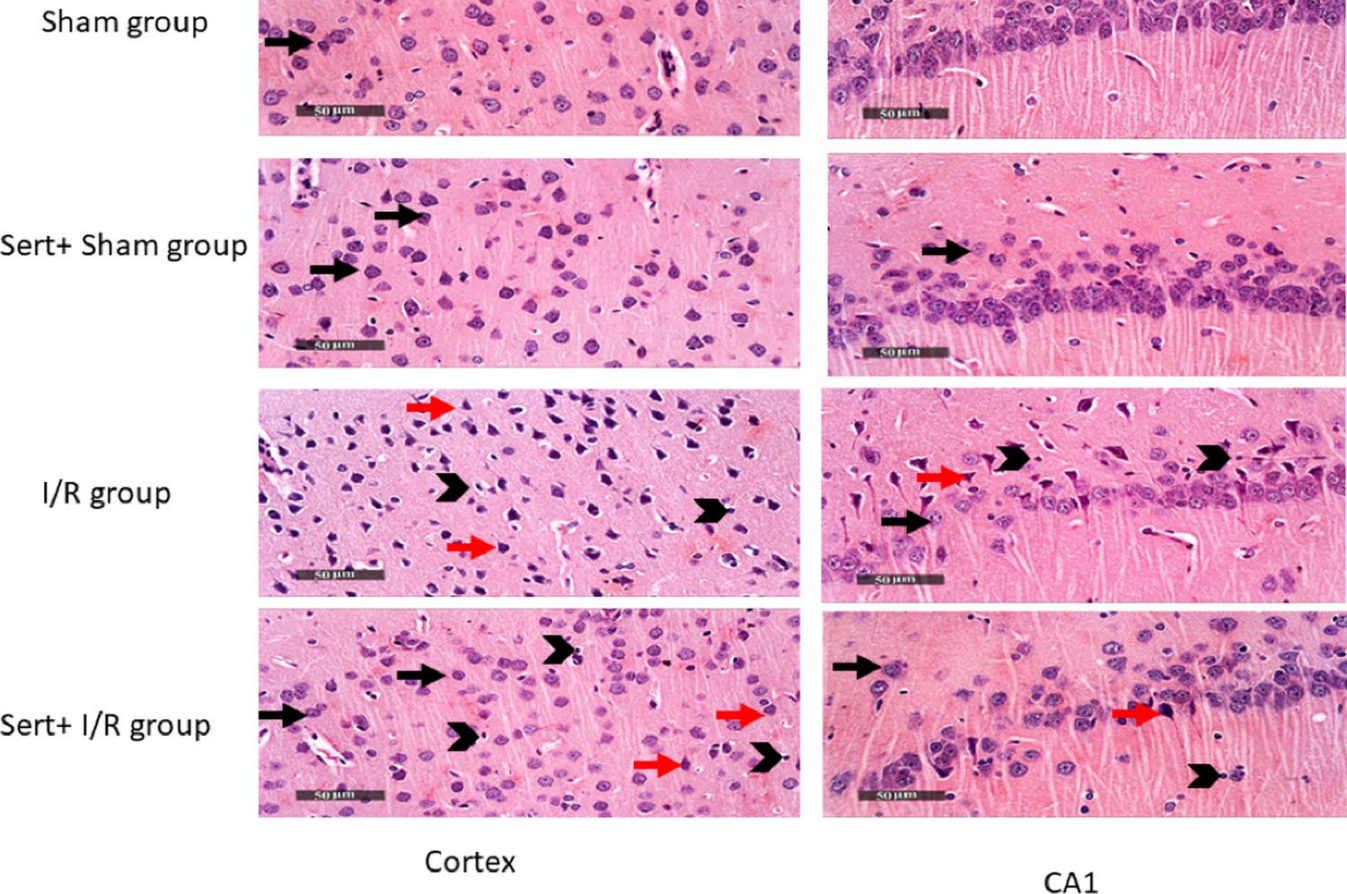
Representative photomicrography showing Sham and Sert cortical samples displaying typical histological features (black arrow). The I/R group showed severe degeneration of neurons (red arrow) and a high degree of reactive glial cell infiltration (arrowhead).On the contrary, pre-treatment with Sert showed attenuation in neuronal damage (red arrow), and many well-organised neurons (black arrow) with milder glial cell infiltrates (arrowhead). Also, Sham and Sert CA1 samples showed standard organised histological features of hippocampal layers (black arrow). I/R group showed many degenerated neurons (red arrow), fewer degrees of intact pyramidal neurons (black arrow) and moderate higher degree of reactive glial cells infiltrates (arrowhead). The pretreated group displayed a few neuronal degenerative changes (red arrow) and many well-organised neurons (black arrow) accompanied with mild infiltration of glial cells (arrowhead). The illustrated photos were magnified by × 400, with scale bars = 50 μm

Sham and Sert cortical samples displayed a standard histological feature of cerebral cortical layers. The I/R group showed severe degenerative neurons and high reactive glial cell infiltration.On the other hand, pre-treatment with Sert showed attenuation in neuronal damage, and many well-organised neurons with milder glial cells infiltrate. Also, Sham and Sert CA1 samples showed typically organised histological features of hippocampal layers arrow.The I/R group showed many degenerated neurons, fewer degrees of intact pyramidal neurons and a moderately higher degree of reactive glial cell infiltrates.The pretreated group displayed a few neuronal degenerative changes and many well-organised neurons accompanied by mild infiltration of glial cells (arrowhead) (Fig. 8).

## Discussion

Cerebral I/R injury is a complicated neurodegenerative disease that leads to serious outcomes and affects individuals’ lives (Wu et al. 2022). The aim of the current was to elucidate the protective effect of Sert against cerebral I/R injury in rats. Oxidative stress is a mainstay pathophysiological mechanism of cerebral injury, which occurs due to the over-liberation of reactive oxygen species (ROS), resulting in an imbalance of oxidant-antioxidant equilibrium in favour of oxidation, and consequently, mitochondrial dysfunction occurs. Mitochondrial dysfunction itself acts as a ROS generator, resulting in a vicious cycle of ROS generation (Wu et al. 2020; Mei et al. 2022).

Consistent with previous studies (Zhang and Cui 2022; Yang et al. 2022), our results showed increased oxidative stress in the cerebral I/R group as manifested by elevated MDA, total nitrate/ nitrite contents and decreased GSH level.

Moreover, inflammation is another deleterious pathophysiological event involved in cerebral deterioration. It is documented that up-regulation of neuroinflammatory mediators in cerebral I/R causes persistent damage to the brain, which plays a crucial role in the injury of neurons occurring in the acute phase of ischemic stroke (Zhao et al. 2020; Nie et al. 2022). This pathway leads to the activation of glial cells through multiple molecular pathways as activated NF-ĸB p65. Glial cells preserve the integrity of the brain tissue and protect it from injury resulting from a dangerous insult. It was stated that microglial cells, representing about 15% of brain cells, are in a resident state until activated by noxious factors as pathogen or neurodegenerative disease. Microglial cells act as a first defence line for CNS microenvironment, which is activated minutes to a few hours following ischemia. In the same context, activation of microglial cells is detected by measurement of Iba-1 expression (Hernández et al. 2021). Additionally, it was found that astrocytes account for 50% of the brain volume. The main function of astrocytes is to maintain the efficient processing of brain tissue by balancing ion-water content, downregulating excitatory neurotransmitters and getting rid of wastes. Astrocyte activation occurs during cerebral I/R, leading to astrogliosis that is detected by GFAP. (Hernández et al. 2021; Jurcau and Simion 2022).

In our study, cerebral I/R resulted in enhanced NF-ĸB p65, GFAP, and Iba-1 protein expression, along with enhanced IL-1, TNF-α levels and decreased IL-10 content. Earlier studies showed that in the cerebral I/R model, oxidative stress enhances transcription factors such as NF-ĸB, which is distributed in the neurons, microglial cells, and astrocytes. This leads to the activation of astrocytes and microglial cells with the subsequent overproduction of inflammatory mediators and a decrease in the production of anti-inflammatory mediators (Zhou et al. 2018; Jurcau and Simion 2022).

The microglial cells activation to classic (M1) or alternative (M2) phenotypes is considered as a macrophage response which occurs due to ischemia. M1 phenotype acts as an indicator of the inflammatory phase by releasing inflammatory cytokines such as IL-1, TNF-α. Many upstream regulatory cascades lead to M1 activation, such as the ERK pathway, activated NF-Kβ p65 and IL-1. Conversely, the M2 phenotype indicates the anti-inflammatory phase, leading to enhanced release of anti-inflammatory cytokines as IL-10 (Zhao et al. 2017).

In the same context, the current data showed a remarkable increase in the expression of ERK, NF-Kβ p65 and CD 86 (M1 marker of glial cells) of the cerebral I/R group. Previous results showed that microglial/microphage polarisation to the proinflammatory M1 phenotype in the cerebral I/R model was attributed to oxidative stress, upregulation of NF-KB and ERK (Gaire et al. 2019; Yang et al. 2019; Jurcau and Simion 2022).

Ischemic stroke can be converted to haemorrhagic stroke due to multiple factors such as ROS and inflammation (Shao et al. 2021; Spronk et al. 2021). During haemorrhagic stroke, lysis of red blood cells occurs. Released haemoglobin (Hb), which participates in the aggravation of oxidative stress, is bound to haptoglobin, forming a complex that binds to CD 163 (a receptor found on neuron cells) and is engulfed by macrophages. Consequently, the upregulation of CD 163 is an indisputable indication of the occurrence of haemorrhagic stroke (Garton et al. 2017). Moreover, a previous study revealed that the expression of CD 163 in neurons is increased after intracranial haemorrhage (ICH) to quarantine the hemolytic product of red blood cells (Hb) and decrease the arising inflammatory cascade as well as generation of ROS. It was found that CD163 expression is increased in both hematoma and perihematomal regions within 6 h after ICH (Garton et al. 2017; Liu et al. 2017).

In our study, Ischemic stroke was transformed into haemorrhagic stroke in the ischemic group, as evidenced by enhanced CD 163 expression in neuron cells. It is noteworthy that haemorrhagic stroke plays a crucial role in maintaining microglial cells in the proinflammatory M1 phase through NF-ĸB-p65 upregulation and ERK phosphorylation (Bi et al. 2021).

In the present study, it was found that levels of MMP-2,9 elevated in I/R group as compared to the sham group. Conversion of ischemic stroke to haemorrhagic stroke was evidenced by measuring matrix metalloproteinase enzymes (MMPs), which have a fundamental role in disrupting BBB via degrading tight junction proteins, resulting in the release of RBCs (Spronk et al. 2021). Previous studies documented that MMP-2 and MMP-9 are essential in BBB disruption during ischemic stroke. It was found that MMP-2 is the initiator of BBB derangement following cerebral ischemia, while MMP-9 is the principal contributor to BBB dysregulation in the delayed phase after ischemic stroke (Lakhan et al. 2013). Moreover, it was demonstrated that upregulated levels of MMPs in the cerebral ischemia group resulted from the generation of reactive oxygen and nitrogen species following reperfusion, leading to haemorrhagic transformation (HT) (Hong et al. 2021). In addition, the cerebral I/R group significantly enhanced HO-2 activity compared to the sham group.

As mentioned before, after leakage, RBCs undergo lysis with the subsequent release of Hb molecules, which are engulfed by the neuronal cells via binding to the neuronal receptor CD163. In the presence of HO-2, an enzyme that is mainly expressed in the neurons, Hb molecules are degraded, leading to the release of intracellular iron, which gives rise to reactive oxygen species (Garton et al. 2017; Liu et al. 2017; Ma et al. 2016). In the same context, as an undisputable confirmation of induction of HT, the Prussian blue stain revealed a significant upregulation of ferric ion deposits in brain tissue as a result of HT in I/R compared to the sham group.

Autophagy plays a severe role in injury caused to neurons due to cerebral I/R. Autophagy is mainly initiated by the liberation of ROS that leads to the downregulation of phosphorylation of PI3K with the consequent inhibition of the downstream mTOR, an essential autophagy regulatory protein. Inhibition of mTOR increases the relative expression of autophagic mediators as LC3 II/I and Beclin-1 (Yan et al. 2019; Yang et al 2019; Zhao et al. 2022). LC3 is present on the autophagosome membrane and is engaged in forming an intact autophagosome. In addition, Beclin-1 is critically involved in lysosome fusion and autophagy initiation (Wei et al. 2021). Moreover, many studies noted that autophagic upregulation is accompanied by apoptotic enhancement (Li et al. 2015; Chen et al. 2022). In our study, PI3K, mTOR, Beclin-1 and LC3 II/I expression were elevated in the cerebral I/R group, along with increased levels of apoptotic makers, caspase-3, Bax, decrease of Bcl-2 content, as well as downregulation of intact neurons number in both cortex and hippocampal CA1 region.

Pre-treatment with Sert exhibited a protective effect against the cerebral I/R model, as shown by decreased MDA, total nitrate/nitrite levels and enhanced GSH content as relevant to the cerebral I/R group. Conforming with our results, a previous study showed that Sert decreased oxidative stress markers in heart failure patients (Michalakeas et al. 2011).

Moreover, Sert down-regulated NF-ĸB-p65, p-ERK, GFAP, Iba-1 expression as well as IL-1, TNF-α contents and increased IL-10 content as relevant to the cerebral I/R group. These outcomes were confirmed by previous studies, which reported that Sert prevented the activation of microglial cells by suppressing NF-ĸB expression (Sitges et al. 2014; Lu et al. 2019).

In addition, pre-treatment with Sert shifted activated microglial cells from the M1 phenotype to the anti-inflammatory M2 phenotype, as detected by increased CD163 and decreased CD 86 expression in comparison with the cerebral I/R group. It was previously documented that M2 polarisation is attributed to many factors, such as decreased activation of ERK, mitigated liberation of ROS and suppressed NF-ĸB-p65 expression (Su et al. 2015; Yang et al. 2019). In the same context, our study showed that Sert inhibits haemorrhagic transformation as detected by suppressed expression of CD163 in neurons, MMP-2,9 levels, ferric ion deposits and activity of HO-2 as compared to the cerebral I/R group.

Finally, compared to the cerebral I/R group, Sert enhanced PI3K and mTOR expression, leading to inhibition of autophagy as indicated by decreased Beclin-1 and LC3II/I expression. Moreover, in consistency with a previous study (Wann et al. 2009), apoptosis was inhibited as demonstrated by the decline of apoptotic markers, Bax, caspase-3, enhancement of anti-apoptotic marker, Bcl-2, and restoration of the number of intact neuron cells. It is worth mentioning that all the aforementioned results were further confirmed by histological examination.

## Conclusion

Our study revealed that Sert has a protective effect against cerebral I/R via multiple pathways, including inhibition of oxidative stress, increased polarisation of microglial cells to the anti-inflammatory M2 phenotype and hence, suppression of inflammation. Moreover, Sert reduced autophagy and apoptosis. Interestingly, our study showed that Sert inhibited haemorrhagic transformation through downregulation of neuronal CD163 expression, attenuation of MMP-2, MMP-9 levels and HO-2 activity.

### Supplementary Information

Below is the link to the electronic supplementary material.

Supplementary file1 (PDF 521 KB)

## Data Availability

All data obtained from this study are presented in the paper. Raw data are available on request.
