# Supplementary figures and images for: Sertraline Pre-Treatment Attenuates Hemorrhagic Transformation Induced in Rats after Cerebral Ischemia Reperfusion via Down Regulation of Neuronal CD163: Involvement of M1/M2 Polarization Interchange and Inhibiting Autophagy
Source: J Neuroimmune Pharmacol. 2023 Nov 13;18(4):657–73. doi: 10.1007/s11481-023-10093-8 (PMC10770270; doi:10.1007/s11481-023-10093-8)

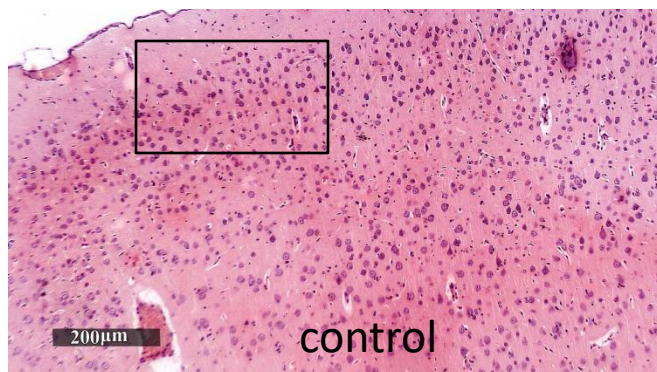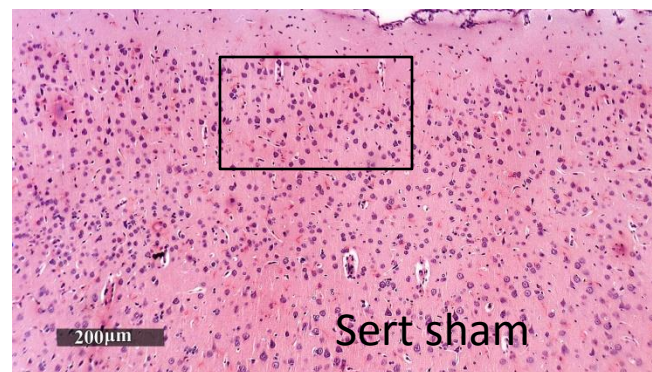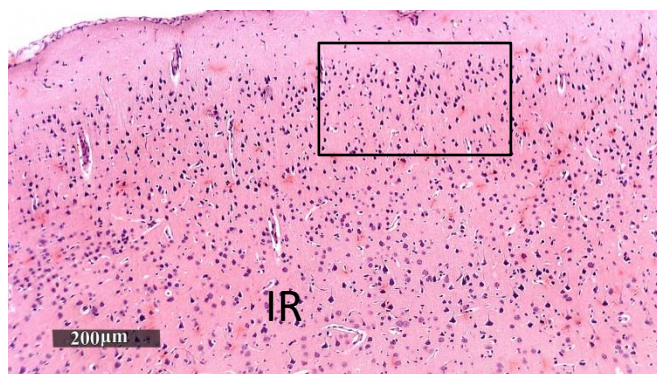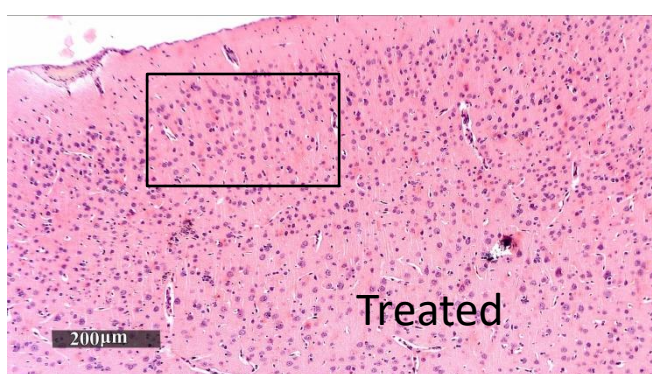

Supplement: Supplementary file 1 — Supplementary file1 (PDF 521 KB) [file 11481_2023_10093_MOESM1_ESM.pdf]
